# Evaluating the impact of a walking program in a disadvantaged area: using the RE-AIM framework by mixed methods

**DOI:** 10.1186/s12889-017-4698-5

**Published:** 2017-09-15

**Authors:** Camila Tiome Baba, Isabela Martins Oliveira, Adriele Evelyn Ferreira Silva, Leonardo Moreira Vieira, Natalia Caroline Cerri, Alex Antonio Florindo, Grace Angélica de Oliveira Gomes

**Affiliations:** 1 Federal University of São Carlos, Washington Luís Highway, 235 km, São Carlos, São Paulo 13565-905 Brazil; 2 University of São Paulo, Doctor Arnaldo Avenue, 715, São Paulo, São Paulo 01246-904 Brazil; 3 University of São Paulo, Arlindo Bettio Street, 1000, São Paulo, São Paulo 03828-000 Brazil

**Keywords:** Process evaluation, Public health, Health management, Physical activity, Aging, Gerontology

## Abstract

**Background:**

The positive health impact of physical activity (PA) is well known, yet a large proportion of the world’s population remains sedentary. General PA programs are common as health promotion initiatives. However, effectiveness evaluations of such PA programs on individual and organizational aspects, which could inform the decision-making process of public health bodies are still lacking, particularly in the most socially disadvantaged areas, where health promotion schemes are particularly needed. The aim of this study was to assess the effectiveness of a Guided Walking Program in a high social vulnerability context.

**Methods:**

A quasi-experimental, mixed methods study was conducted. The program had a duration of 6 months and a 6-month follow-up period after the intervention. Session frequency was five times a week, where sessions consisted of supervised PA combined with educational sessions. The Reach, Effectiveness, Adoption, Implementation and Maintenance (RE-AIM) framework was followed to assess the program. The International Physical Activity Questionnaire (IPAQ) and accelerometers were used to measure levels of PA. Focus groups were conducted to gain a comprehensive insight on the implementation domain.

**Results:**

Most subjects in the intervention (IG) (n = 74) and control (CG) (n = 74) groups were female (IG:90.5%; CG:95.9%), aged 18–49 years (IG:44.6%; CG:43.2%), received less than 1 minimum wage (IG:74.3%; CG:83.7%) and had 0–4 years of formal education (IG:52.1%; CG:46.1%). The reach of the intervention was 0.3%. The IG showed increased levels of PA at post-intervention and 6-month follow-up. However, the difference between groups was not statistically significant. Adoption data revealed that 89.5% of the professionals in the Primary Health Care Center (health center) team perceived the benefits of the program for the population. The program was independently promoted by the health center team for a further 4 months post-intervention. The qualitative data revealed that the program was discontinued due to participants’ low adherence and human resource limitations in the unit’s operational dynamics.

**Conclusions:**

A health promotion intervention in a socially deprived setting faces challenges but can be effective and feasible to implement. The present study informs the development of future health promotion initiatives in this context.

**Trial registration:**

NCT02857127. Registered: 30 July 2016 (retrospectively registered).

## Background

According to the World Health Organization [1], approximately 23.3% of the world population and 32.4% of the American population are physically inactive. An estimated one in every three adults is sedentary, and physical inactivity may increase the risk of death by 20% to 30%. [2, 3]. The cost of physical inactivity to the world’s economy is estimated at $53.8 billion [4–6]. In Brazil, the percentage of individuals practicing less than 150 min of moderate PA per week was 47.5% while 16% reported practicing no PA in the past 3 months [7].

Offering physical activity programs can help change this scenario worldwide [8–10]. Low-income individuals, higher age groups and females are the groups most vulnerable to becoming physically inactive [11], pointing to the need for greater investment in health promotion interventions in this population.

Disparities among socioeconomically different areas may affect the applicability of PA programs. Environmental factors, lower absolute income, greater population density, lack of safety and accessibility in high social vulnerability areas may all negatively impact the practice of PA [12–14]. Therefore, it is essential that the nature of these challenges is well understood and the effectiveness of different approaches for promoting PA is objectively measured.

According to Wierenga [15], the heterogeneity of evaluations of health promotion programs hampers comparison of these initiatives, and poses a major barrier to correlating effectiveness and implementation. Thus, it is important that program implementation and its effectiveness analysis are clearly reported to allow comparison across studies [16]. Moreover, it is relevant to conduct studies exploring external validity of interventions, currently little investigated in the scientific literature [17, 18].

The Reach, Effectiveness, Adoption, Implementation and Maintenance (RE-AIM) framework is a complete, global instrument for identifying individual and organizational characteristics of health interventions [19] and has been widely utilized in international studies [20–23]. A recent systematic review on the use of RE-AIM identified the need for further studies analyzing other operational aspects in addition to intervention effectiveness [23]. In developing countries, particularly in high social vulnerability areas, few studies have employed the RE-AIM to evaluate health promotion interventions, where greater use of this tool may enable comparison of health promotion programs in different contexts. Results of PA programs assessed with this tool allow proper planning of these interventions and enhancement of methodologies [10, 24]. The aim of this study was to assess a Guided Walking Program (program) in a high social vulnerability context, using the RE-AIM framework.

## Methods

A controlled, mixed methods, quasi-experimental study was conducted. Data collection occurred between July 2014 and July 2016 at Primary Health Care Centers (health centers) in the City of São Carlos, São Paulo State, Brazil.

The selected areas for data collection were two regions that had the highest Social Vulnerability Index of the State of Sao Paulo (Paulista Index of Social Vulnerability) [25]. The target-population consisted of individuals registered at five health centers in the two areas selected for the study. The two areas, for intervention (IG) and control (CG) groups, respectively, were geographically distant to minimize the risk of bias.

A total of 195 individuals took part, comprising 88% women, and with a mean age of 47.8 (± 12.7) years. For data analysis, the inclusion criteria for both groups were: 18 years of age or older; able to understand the questions; agreeing to participate in the study; and at the “insufficient PA level” (i.e., practicing less than 150 min of PA per week, as measured by the International Physical Activity Questionnaire) [11]. This project was approved by the Ethics Committee of the Federal University of São Carlos (number 384.852).

### Intervention

The intervention was carried out by the health centers in partnership with the researcher team and consisted of five weekly health promotion sessions for 6 months. The health promotion sessions included supervised PA combined with educational initiatives. The PA component consisted of 4 days of a 50-min walk and 10 min of stretching/warm-up, and 1 day of varied exercises such as Zumba, stretching and recreational activities. The intensity of the walk was controlled with a Polar FT1 Heart Rate Monitor and by the colored version of the Borg scale measuring perceived exertion of the participants [26]. Participants were instructed to maintain a moderate and/or vigorous intensity of the PA. All classes were supervised by physical education professionals together with the professionals from the health centers and took place in the vicinity of the centers. As a preventive health procedure, blood pressure was measured every session. The weekly frequency and duration of the activities followed the guidance provided by Garber et al. [27].

The educational initiatives took place immediately after the PA component and included the following: 1) Small interactive activities, debates and/or lectures of 10 to 15 min on alternating weeks, focusing on strategies for behavioural change and incentives to incorporate PA into daily life. 2) Daily health tips for 5 min about important behaviors for a healthier lifestyle when practicing PA, such as drinking fluids, applying sunscreen, and wearing appropriate footwear. Additionally, health topics of interest to the group and health promotion themes proposed by the Brazilian Department of Health calendar [28] were discussed.

Motivation strategies to promote adherence to the program were applied throughout the 6-month intervention. Prizes were awarded to those who missed the least number of sessions every month. Example prizes included fruit baskets and sports accessories such as gym towels and gym bottles. Social relationships were also strengthened by stimulating conversation among the participants during the walking sessions. Phone calls were made to those who missed 3 days in a row without justification.

The CG received no information about physical activity recommendations during the three PA level-related assessments.

### Data collection

Data collection for both groups was carried out at three timepoints: prior to the intervention (T0), immediately post-intervention (T1) and after a 6-month follow-up period (T2).

Sociodemographic data, including age, sex, education, as well as individual and family income, were collected at baseline (T0).

The RE-AIM framework was used to assess the intervention. This tool was validated in Brazil by Almeida, Brito and Estabrooks in 2013 [24], and showed satisfactory internal and external validity [17, 19]. The RE-AIM instrument has been widely utilized in the literature [17, 20–23] and consists of five main components: reach, efficacy, adoption, implementation and maintenance. Each of these components is outlined in greater detail below.

### Reach

“Reach” measures the dimension and coverage of the intervention in the community [24]. This component was determined by calculating the proportion of participants on the first day of the program divided by the number of people who possibly became aware of the program through various forms of advertising. This study used the following advertising strategies: health center teams were asked to refer patients; banners were displayed in the neighborhood; a car broadcasting an audio message in the neighborhood; door-to-door distribution of invitations and personal invitations within health center waiting rooms. Social media and a website containing information about the program were also used to promote the program during the 6-month intervention. Most of the streets in the vicinity of the health centers were covered by the advertising strategies.

### Efficacy or effectiveness

This component evaluates the individual effectiveness of the intervention [17]. Changes in levels of PA were measured using the International Physical Activity Questionnaire (IPAQ), validated for use in Brazil [29], collecting PA time in minutes per week in the leisure time domain and the sum of all domains (total PA). An accelerometer (Actigraph GT3X) was used to calculate the number of counts per minute per day in an average week. The volunteers wore the device around the waist for 8 consecutive days. The data collected were analysed by the software ActiveLife 6.8 version, employing an epoch of 1 s and the “daily” algorithm. The wear-time required for inclusion was 9 h for at least 4 days a week, including at least one weekend day. The cut-offs proposed by Freedson [30] were used in the Active Life software. Both of these evaluations of PA level were performed at T0, T1 and T2.

Individuals who accomplished less than 150 min of leisure time PA (LTPA) a week on the IPAQ were classified as “insufficiently active”.

### Adoption

To analyze organizational involvement in the intervention [24], a five-item questionnaire was applied collecting information on the direct or indirect involvement of all professionals with the IG at the health centers. The questionnaire was left at the centers for 15 days and the professionals were instructed to answer them anonymously and voluntarily.

### Implementation

“Implementation” measured the extent to which the project was completed as planned. Two forms of measurement were used to grasp “implementation” both at organizational and individual levels.

A questionnaire containing the essential items of the project was devised. This questionnaire was answered by adherent participants (>80% participation) and those most involved from the health center teams (as perceived by all members involved in the program). The questions collected data on the number of participants in the intervention; the Borg scale and Heart Rate Monitor; blood pressure measurements before the activities; project advertising for 2 weeks; the several forms of advertising (sites, folders, banners, personal invitations, home visits within the community, website); counseling for diabetics; monthly blood glucose measurements; and the offering of educational initiatives.

In addition, a focus group was conducted at the end of the intervention to analyze information on program implementation. A previously trained mediator applied the focus group techniques, forming a small group of participants and professionals with greatest adherence to the program. The mediator did not participate at any time in the program, and was given a checklist about the essential items of the program divided into four dimensions: dissemination, adherence, class protocol and assessments.

### Maintenance

To assess this domain on the organizational level, continuity of the program was checked at the health centers. Structural conditions (human and physical resources) contributing to the maintenance of the program were explored in structured interviews. The questions asked were: 1) Did the Project continue after the end of 6-month intervention when the research team withdrew?; 2) If not, what were the main difficulties?

On the individual level, participants’ PA level was analyzed after 6 months of intervention by the IPAQ and the accelerometer using the same methodology applied for the effectiveness domain. Behavioral changes were determined, by comparing these data with T0 and T2, as well as against the CG.

### Data analysis

#### Quantitative data

Descriptive analysis was carried out using absolute and relative frequency measures for categorical variables, and mean, standard deviation and median for numeric variables. Data were analyzed using the Statistical Package for the Social Sciences (SPSS) software, version 22.0. Data normality was verified by the Kolmogorov-Smirnov test. The Chi-square test was used to compare categorical data between the IG and CG. Generalized estimation equations with the inverse Gaussian distribution function were used for non-parametric data. The normal distribution function and the identity link function were used for the PA scores. For the weekly and daily PA variables, the inverse Gaussian distribution function and the identity binding function were used. For these variables, 1 min per week or 1 min per day was added to all values ​​to avoid null values ​​(values ​​must be >0 in an inverse Gaussian distribution). In all cases, an unstructured correlation matrix was used to independently estimate each variance and covariance, while the Huber-White estimator was used to account for the possible heteroskedasticity resulting from the calculation of standard errors. For each outcome, the effect of belonging to a given group, the time elapsed since the beginning of the intervention (T0, T1 and T2), and the interaction of these two factors were estimated to evaluate possible differences in the temporal trends of PA between the groups.

The Bonferroni’ test was used for multiple post-hoc comparisons [31, 32]. A statistically significant difference for values of *p* < 0.05 was adopted.

#### Qualitative data

The focus group was formed to analyse the implementation domain. The discourse of all participants in the group was sound recorded and transcribed after previous consent. Subsequently, inferences and interpretations were analyzed by dimension following three steps: data pre-analysis, content exploration and treatment of results [33, 34]. Some key excerpts of discourse were highlighted for reporting in the results.

## Results

### Sample selection and recruitment

**Fig. 1:**
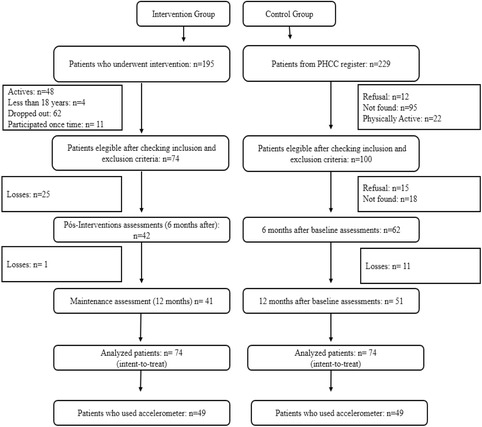
Flowchart of participant loss

For the IG, a total of 195 users of the health centers participated in the program. Of the total participants, 74 users met the inclusion criteria and agreed to take part in the study. For the CG, 299 registered users of a health center located in another high vulnerability area of the city not part of the project were selected. Of this group, 100 service users met the inclusion criteria. The “not found” for the CG refers to individuals that had moved or for whom the research team did not have access to the new address or who were not found after 3 visits. The CG was matched with the IG for gender and age, giving 74 individuals for inclusion in the CG. Participant losses (Fig. 1) were determined by the imputation method of missing data and intention-to-treat.

### Sociodemographic and economic characteristics

Sociodemographic and economic characteristics of the groups were similar (Table 1). Both groups consisted predominantly of women (IG: 90.5%; CG: 95.9%), individuals aged 18 to 49 -years (IG: 44.6%; CG: 43.2%), with more than 4 years of formal education (IG: 61.6%; CG: 77.8%), a family income higher than 1 minimum wage (IG: 83.8%; CG: 70.3%) and an individual income of up to one minimum wage (IG: 73.6%; CG: 83.8%).

**Table 1:** Sociodemographic characteristics (%) and comparison of intervention and control groups attended in primary health care. São Carlos, SP, Brazil, 2015

Sociodemographic characteristics	Intervention *n* = 74	Control n = 74	p – value^a^
	%	%	
Age group (years)			
18–49	44.6	43.2	0.979
50–59	31.1	31.1	
≥ 60	24.4	25.7	
Gender			
Female	90.5	95.9	0.327
Male	9.5	4.1	
Formal education (years)			
0–4	52.1	46.3	0.785
5–8	26.7	28.9	
≥ 9	21.2	24.8	
Individual income (minimum wages)			
< 1	74.3	83.7	0.156
≥ 1	25.7	16.3	
Family income (minimum wages)			
< 1	16.3	29.7	0.051
≥ 1	83.7	70.3	

^a^ *Chi-square Test*

### RE-AIM dimensions

#### Reach

The reach of the project was 0.3%. This was calculated as a ratio of the number of participants on the first day of the program (*n* = 27) to the number of people who most likely heard about the program (*n* = 7480). This latter number was calculated based on the number of houses on the streets in the vicinity of the health centers and the mean number of residents per household (three).

#### PA level (effectiveness/efficacy and maintenance)

**Fig. 2:**
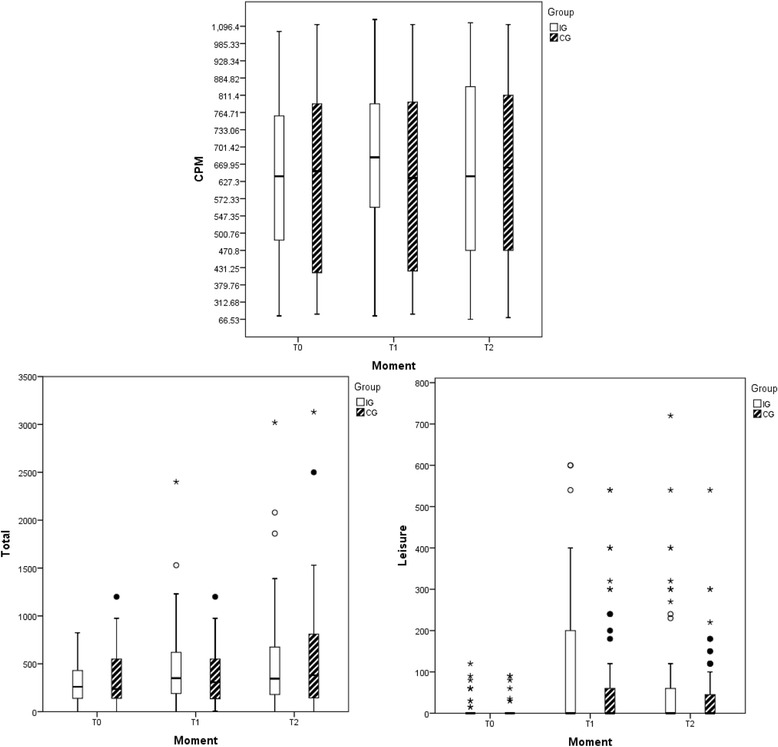
Levels of PA according to accelerometer (counts) and IPAQ domains (total and leisure time)

The descriptive analyses of the groups for the three timepoints are depicted in the Box-Plot (Fig. 2). The comparative analyses according to group, timepoint and interaction between group and timepoint are shown in Table 2. A significant difference in Total PA and Counts per minute was found for the group and timepoint interaction (*p* = 0.032; *p* = 0.014). Both groups gradually increased PA practice from T0 to T2 in LTPA, Total PA and Counts per minute, with the exception of the CG for LTPA (T0–52.8; 95%CI = 32.6–73.0, T1–73.0; 95%CI = 46.0–99.9; T2–71.3; 95%CI = 53.5–89.2) and the IG for Counts per minute (T0–638.4; 95%CI = 580.5–696.3; T1–693.9; 95%CI = 629.3–758.6; T2–655.4; 95%CI = 591.4–719.3). Despite a decrease in minutes per week between T1 and T2, LTPA remained higher than at baseline (T0).

**Table 2:** Estimated average (95%CI) physical activity level of adults and older adults attended in primary health care and comparative analysis. São Carlos, SP, Brazil. 2015

		Baseline (T0)	6 months (T1)	12 months (T2)	p-group^a^	p-timepoint ^b^	p-interaction^c^
Leisure time physical activity (LTPA)	IG	52.1 (33.4–70.8)	71.7 (44.2–99.2)	84.3 (62.2–108.3)	0.752	0.003*	0.788
	CG	52.8 (32.6–73.0)	73.0 (46.0–99.9)	71.3 (53.5–89.2)			
Total Physical Activity (PA)	IG	303.2 (253.7–352.7)	450.5 (360.2–540.7)	501.4 (382.9–619.9)	0.712	<0.001*	0.032*
	CG	332.1 (270.8–393.5)	349.6 (289.6–409-6)	525.3 (401.6–649.5)			
Counts per minute	IG	638.4 (580.5–696.3)	693.9 (629.3–758.6)	655.4 (591.4–719.3)	0.478	0.025*	0.014*
	CG	623.8 (564.6–683.1)	622.9 (565.6–680.1)	656.4 (597.8–714.2)			

*p < 0.05

^a^Comparison of mean for group, independently of timepoint

^b^Comparison of mean for timepoint, independently of group

^c^Values for interaction between timepoint and group

^d^p-value estimated by Generalized estimation equations

#### Adoption

A total of 19 questionnaires were fully completed and analyzed for the “Adoption” domain. This figure represented 59.3% of the professionals working in the health centers involved (Table 3).

**Table 3:** Health professionals´ perceived involvement in the program. São Carlos, SP, Brazil

I feel involved with the implementation of the program.
63.1% Agree
26.3% Partially agree
10.6% Partially disagree
0% Disagree
I see the benefits the program brings to the community.
89.5% Agree
10.5% Partially agree
0% Partially disagree
0% Disagree
Does the team comment about the walking program?
94.7% Yes
5.3% No
I referred people for the program.
100% Yes
0% No

The vast majority of responses were positive. Notably, most of the team commented about the program, and all the professionals that answered had referred individuals for the program. Also, the majority of respondents saw the program benefits.

#### Implementation

The questionnaires were applied to two Community Health Workers and three participants involved in the intervention (Table 4).

**Table 4:** Checklist for the implementation domain

Percentage	Item
80%	Report having observed more than 44 participants of the group that met the inclusion criteria
20%	Remember maintaining the intensity of exercises using the Borg scale
100%	Report having used the Heart Rate Monitor
100%	Recall there was blood pressure measurement before the activities
80%	Believe the advertising campaign occurred for 2 weeks
60%	Remember seeing all modalities of advertising: sites, folders, banners, personal invitations, home visits within the community and program websites
80%	Remember there were orientation sessions for diabetics
40%	Report the team requested glycaemia checks 1× a month
100%	Remember the application of educational initiatives

It should be noted that, although only a few individuals reported the monitoring of exercise intensity using the colored Borg scale, all remembered using the Heart Rate Monitor, which had the same function.

The focus group found that, regarding the program advertising campaign, the participants reported a range of channels that made them aware of the study: “I heard of it through the internet, on Facebook (…) And the advertising car also passed by.” (Participant 1).

The active participation of health center professionals was also a key element in advertising the program: “What we did was: each professional had their own coverage area to take care of, which involved communicating mainly with individuals such as Participants 2 and 3. So the team went ahead and handed out flyers.” (Community Health Workers 1).

Regarding group adherence to the program, fluctuation in the number of participants was evident (Participant 2: “Some days there are 40, some days there are 30 something.”). The strategies for improving adherence were also noted (Participant 1: "The teacher will also call to find out what happened and the staff will let you know when they are going to be absent."). The target-population was of the age proposed (Participant 3: "A lot older than 20 years.").

As for the class protocol and monitoring measures, several aspects were highlighted including how the classes developed, as well as the materials used. When asked about the pressure blood check, warm-up and stretching, all participants reported these were done every day. The devices used were also highlighted (Participant 3: "That little device here (abdomen) and on the arm too."; Participant 2: “It’s called a pedometer.”). Comments were also made on the devices for maintaining PA intensity and verbal orientations: “They encourage you to push a little. Feeling a little breathless, otherwise it does not work. Just walking around does not work, you have to be panting.” (Participant 1).

The educational initiatives were also highlighted: “At least three times a week there was some sort of orientation. (…) About diseases too. Breast cancer, for men also prostate. They always had these orientation sessions.” (Participant 1).

Finally, for health assessments, the evaluation protocol followed by participants and anthropometric measurements were mentioned (Participant 1: We were weighed, took measurements and had a questionnaire. Also about memory. A very thorough evaluation. And I’ve been through the evaluation again.)

#### Maintenance (organizational level)

The program was independently promoted by the health center teams for a further 4 months post-intervention. After these 4-months, based on the health professionals´ report, it was decided to suspend the Project due to the increased workload burden and low attendance rate of the sessions, as well as infrastructure and human resource limitations in the centers’ operational dynamics. The professionals also reported a lack of time to execute and lead the Project considering their other operational activities in the health centers. Moreover, the low participant adherence immediately after the 6-month intervention led to demotivation of the health professionals who decided not to continue the activity.

## Discussion

The program aim was to promote regular PA in the long term within a socioeconomically deprived context. The program was found to be effective and had adequate involvement of the health center professionals but low adherence post-intervention.

This study was a pioneering investigation in Brazil since previous research has focused on non-deprived areas [35–38], applied different interventions [35–39] and used other measurement tools [37, 39]. In contrast to the present investigation, previous studies assessed interventions in more generic contexts, involving different target-publics or failed to analyze mixed educational and supervised intervention. Brazilian studies that utilized the same tool as the present study analyzed the university environment or analyzed interventions involving only educational initiatives for behavioral change [35, 40, 41].

Only one quarter of the residents that accepted the invitation to participate in the intervention were considered active, showing a lower PA level than the general Brazilian population [42]. Locations with the highest poverty indexes are considered to be those that have the greatest difficulty accessing health promotions and poor information relative to other regions [43–45]. This investigation involved individuals who predominantly had a low educational level, low income, female gender and older age. The elderly are a group more prone to physical inactivity and, thus, in need of more accurate information concerning their health [10, 11]. Thus, the study results are congruent with the goals of policies promoting a more active lifestyle to reduce costs in health services, especially among socially vulnerable individuals [4, 28, 46, 47].

Adherence remained a challenge. Despite wide advertising prior to the beginning of the program, the reach was low, differing from other studies [22, 47, 48]. It is important to emphasize that a large proportion of residents in the area studied worked full-time and travelled to work at the time of the intervention, a factor that could possibly explain the low adherence levels and high number of dropouts from the program. Moreover, the “Reach” domain of the RE-AIM framework is measured on the first day of the intervention [24], which does not reflect the actual number of subjects who underwent the program over time. Future studies should consider this information when applying interventions in disadvantaged areas.

However, positive results were found regarding effectiveness and maintenance. The IG considerably increased PA levels, including during the follow-up period. In terms of public health gains, despite the recommendations of at least 150 min of weekly moderate PA, the increase of at least 10 min of weekly PA for a large percentage of the participants is a highly relevant result, given that the prevalence of physical inactivity and sedentary behavior continues to rise, and the number of insufficiently active or inactive subjects (< 10 min of PA a week) in Brazil remains high [49].

Analysis between groups did not identify statistically significant differences in increased PA levels over time. The study performed three home assessments using a questionnaires on PA level and made three requests to use the accelerometer for a week. These assessments may have encouraged volunteers of the CG to reflect on their health behaviors. Other studies involving interventions for vulnerable groups also showed an increased PA level in the control group, even in randomized interventions [50, 51]. This fact demonstrates a possible “assessment as intervention” effect, evidencing the importance of the population’s participation in scientific studies.

On the organizational level, it was reported that the involvement of health center teams was successful in this context. The level of involvement of professionals in these teams was indeed better than expected, because a large number of these professionals reported experiencing burnout, overload and high demand of administrative activities, which would typically deter them from becoming more involved in group activities directed toward health prevention and promotion [39, 52–54]. This result may be attributable to the positive perception reported by the professionals in the focus group regarding the adequate functioning of the program. This must be considered when applying similar studies in the future.

The study also expected to find difficulties for the health centers to continue the program immediately after our team left. However, this problem occurred only after 4 months. Importantly, a lack of resources plus the absence of a specialized professional to apply PA interventions may have affected the continuity of the program [54–56]. Furthermore, the low-income population’s understanding about the role of all the professionals involved in primary care and the prevention and promotion of health should be strongly reinforced since the adherence of participants decreased after withdrawal of the research group.

Regarding the implementation domain, despite the structural limitations of the location, in general, the research team was able to accomplish the proposed activities in their work plan. The results were better than those reported in some other health intervention studies that utilized the RE-AIM framework as an evaluation tool [23, 57]. The challenges of rigorously following the application protocols of an intervention occur due to the dynamic process in which this takes place over time.

The non-randomized design, low population adherence, location structural barriers, as well as the high level of dropouts, were considered relevant limitations to the analyses of the present study. Although the sample was selected by convenience, the groups may have had different characteristics, but were similar for sociodemographic characteristics and level of physical activity before starting the program.

Future studies should address these limitations, but continue to measure more than one type of evaluation of the RE-AIM domains to ensure sensitive analysis of all variables. This would generate a greater wealth of details for the discussion and results. For a more thorough intervention assessment, a cost analysis is recommended in order to facilitate applicability of these interventions in practice and enable comparison of cost-effectiveness of different interventions.

## Conclusions and implications

We can conclude that the analyses of individual and organizational levels of the program performed using the RE-AIM framework showed positive results of program participation, including the health professionals of the health centers. Therefore, given the challenges faced, such as infrastructure and low-income population behavior characteristics, the program was considered effective.

This study was carried out in a disadvantaged area in Brazil. Despite specific differences in disadvantaged areas in other Brazilian states and poor countries, we believe that low income, low education and poor living conditions are similar among all impoverished areas. Therefore, this study may serve as a model for further studies which should continue to explore the challenges that social vulnerability areas represent in terms of health promotion.

## Abbreviations

CG: Control group
IG: Intervention group
IPAQ: International Physical Activity Questionnaire
LTPA: Leisure-time physical activity
PA: Physical activity
