# Correction to: Evaluating the impact of a walking program in a disadvantaged area: using the RE-AIM framework by mixed methods

**DOI:** 10.1186/s12889-017-4826-2

**Published:** 2017-10-18

**Authors:** Camila Tiome Baba, Isabela Martins Oliveira, Adriele Evelyn Ferreira Silva, Leonardo Moreira Vieira, Natalia Caroline Cerri, Alex Antonio Florindo, Grace Angélica de Oliveira Gomes

**Affiliations:** 1 Federal University of São Carlos, Washington Luís Highway, 235 km, São Carlos, São Paulo 13565-905 Brazil; 2 University of São Paulo, Doctor Arnaldo Avenue, 715, São Paulo, São Paulo 01246-904 Brazil; 3 University of São Paulo, Arlindo Bettio Street, 1000, São Paulo, São Paulo 03828-000 Brazil

## Correction

After publication of the article [1], it has been brought to our attention that the full funding acknowledgement is missing from the original article. It should read as below –.

This study was financed by the Fundação de Amparo à Pesquisa do Estado de São Paulo (FAPESP) (grant nº 2014/17419–7). Alex Antonio Florindo receives a fellowship from the Brazilian National Council for Scientific and Technological Development (CNPq) (grant 306,635/2016–0).
